# Agri-Food Biowaste Bioactives for Biopesticides: A Circular Economy Solution with Industry 4.0?

**DOI:** 10.3390/molecules31060996

**Published:** 2026-03-16

**Authors:** Thiago F. Soares, Rita C. Alves, Maria Beatriz P. P. Oliveira

**Affiliations:** LAQV-REQUIMTE, Faculty of Pharmacy, University of Porto, Rua de Jorge Viterbo 228, 4050-313 Porto, Portugal; beatoliv@ff.up.pt

**Keywords:** phytochemicals, green chemistry, plant protection, by-products valorization, sustainable agriculture

## Abstract

The widespread use of synthetic pesticides has ensured crop productivity but has also raised serious environmental and human health concerns, including water contamination, biodiversity loss, and intoxication risks. In this context, global strategies for sustainable agriculture, safer alternatives are urgently needed. This systematic review, conducted in accordance with PRISMA guidelines, examines the potential of agri-food by-products as sources of bioactive compounds for biopesticide development within a circular economy framework. Residues from major agri-food chains, including the olive, potato, banana, citrus, and winery industries, were systematically analyzed with respect to their phytochemical composition, such as phenolics, flavonoids, terpenoids, fatty acids, and essential oils, and their reported bioactivity against insects, weeds, fungi, bacteria, and nematodes. The mechanisms of action, technological recovery strategies, and formulation challenges are critically discussed. Additionally, regulatory challenges and opportunities in the European and U.S. markets are described together with the role of Industry 4.0 technologies in optimizing recovery processes and product development. By promoting biopesticides from agri-food biowaste, this approach contributes to sustainable production (SDG 12), innovation in industrial processes (SDG 9), and the protection of terrestrial and aquatic ecosystems (SDGs 14 and 15), positioning food industry residues as a strategic resource for green crop protection.

## 1. Introduction

**Figure 1:**
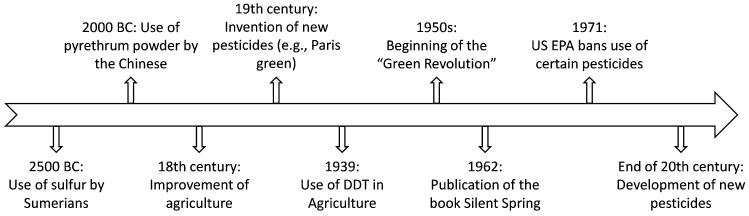
Timeline of pesticide history.

In order to enhance living conditions through agricultural practices and food storage, chemical substances have been developed to increase production and mitigate food loss [1]. Their use has been documented since Classical Antiquity (≈2500 BC) and was not solely an invention of the modern chemical industry, as illustrated in Figure 1 [1]. The 19th century was of paramount importance for pesticide development, owing to the emergence of various substances, primarily inorganic ones [2]. A major milestone occurred in 1939, with the confirmation of the insecticidal action of dichlorodiphenyltrichloroethane (DDT), demonstrated in 1939 by the Swiss chemist Paul Müller [3,4].

Following World War II, in the 1950s, Europe faced severe food shortages due to extensive destruction of fields and pastures, initiating a period of intensive use of these substances for rapid food generation, termed the Green Revolution [2,5]. However, in 1962, Rachel Carson highlighted the environmental and human health issues caused by pesticide use [6]. As a result, numerous studies were conducted regarding pesticides and their toxicity. Starting in 1971, the United States Environmental Protection Agency (US EPA) banned or restricted several synthetic pesticides [4]. Synthetic pesticides are generally non-selective and have a bioaccumulative nature, targeting specific biochemical reactions that are common to many living beings, leading to secondary effects [5]. Baird and Cann 2012 [7] showed that DDT bioaccumulates across trophic levels, with high concentrations found in osprey adipose tissue due to their fish diet, which contains DDT that is millions of times higher than in plankton. Over the past decade, the agrochemical sector has introduced several structurally distinct active ingredients designed to improve resistance management and enhance biological selectivity. Among these, flupyradifurone, a butenolide insecticide, interacts with nicotinic acetylcholine receptors through a binding profile that differs from classical neonicotinoids, contributing to its effectiveness against resistant insect populations [8]. Similarly, broflanilide, classified as a meta-diamide, acts as a non-competitive modulator of γ-aminobutyric acid (GABA)-gated chloride channels at a novel site, representing a new mode of action within insect control strategies [9]. In the field of fungicides, oxathiapiprolin targets oxysterol-binding protein-related pathways in oomycetes, demonstrating remarkable potency at very low application rates [10]. Benzovindiflupyr, a succinate dehydrogenase inhibitor (SDHI), interferes with mitochondrial respiration and was developed to provide extended residual activity and improved metabolic stability [11]. These examples illustrate how contemporary synthetic pesticides increasingly rely on molecular-level specificity and innovative target sites to address resistance and maintain crop protection efficiency.

Pesticides impact human health through acute or chronic intoxications during handling or by consuming contaminated food [12]. The World Bank reports that approximately 355,000 deaths occur annually worldwide due to pesticide poisoning [13]. Investigations conducted by do Nascimento et al. 2017 [14] regarding the effects of organochlorines on populations that are chronically exposed to them revealed adverse effects on the liver and hormonal alterations, also detected in donors’ blood.

Despite the existence of various measures to mitigate environmental risks and human health concerns, the rational and sustainable use of pesticides remains one of the greatest challenges for large-scale sustainable agriculture [15]. Governmental commitment is required through improvements in policies related to the use of such compounds, such as the potential growth in the use of biopesticides, as observed in the documents Towards a Sustainable Europe by 2030 from the European Union (EU) and the Sustainable Development Goals (SDGs) of the United Nations (UN) 2030 Agenda [16]. With the projected global population reaching 9.8 billion by 2050, the demand for food will increase substantially [17]. At the same time, efforts to reduce the reliance on synthetic pesticides pose a challenge for farmers, as they must maintain crop yields while managing pest populations [17]. Excessive use of the remaining available products can accelerate the development of pest resistance, reducing their long-term efficacy and increasing the need for alternative pest management strategies [17].

Due to the adverse effects of synthetic pesticides, they are being replaced by less impactful alternatives or natural products. The reviewed studies indicate that by-products from agri-food industry processing represent promising sources of biologically active compounds with high potential to become significant raw materials for obtaining biopesticides [18,19]. Accordingly, this systematic review aims to critically analyze the potential of bioactive compounds extracted from agri-food industry waste as biopesticides, emphasizing their chemical diversity, biological activity, and relevance within circular economy and sustainable agriculture frameworks. It is important to acknowledge, however, that a natural origin does not automatically guarantee safety. Several plant-derived molecules may exert toxic effects on non-target organisms, including beneficial insects, aquatic species, and even mammals, depending on the concentration and exposure conditions. Therefore, although biopesticides obtained from agri-food residues are frequently associated with improved environmental compatibility, their development and practical application must rely on comprehensive toxicological and ecotoxicological evaluation, as well as the assessment of environmental persistence and unintended biological effects. A critical evaluation of both efficacy and potential risks is essential to ensure that these alternatives effectively contribute to sustainable agriculture.

## 2. Methodology

This systematic review was conducted by following structured methodological principles inspired by the Preferred Reporting Items for Systematic Reviews and Meta-Analyses (PRISMA) guidelines to enhance transparency and reproducibility.

### 2.1. Literature Search Strategy

A comprehensive literature search was performed in the Scopus, Web of Science Core Collection, and PubMed databases between January and March 2025. The search strategy combined controlled vocabulary and free-text terms related to agri-food waste, including “pesticide”, “biocide”, “biopesticide”, “bioherbicide”, “biofungicide”, “bioinsecticide”, “phenolic compounds”, “fatty acids”, “essential oils”, “by-products”, “bio-wastes”, “agri-food industry”, “olive”, “citrus”, “banana”, “circular economy”, and “industry 4.0”. Boolean operators (“AND”, “OR”) were applied to refine search combinations.

Although the primary focus was on studies published after 2000, selected earlier publications were included due to their unique contributions to the field.

### 2.2. Eligibility Criteria

The inclusion criteria were defined as follows:

(i) Peer-reviewed original research articles or review papers;
(ii) Studies reporting the extraction, identification, or characterization of bioactive compounds derived from agri-food industry residues;
(iii) Investigations evaluating pesticidal, insecticidal, fungicidal, herbicidal, or antimicrobial activities that are relevant to agricultural applications;
(iv) Studies providing experimental data, mechanistic insights, or quantitative biological assessment.

The exclusion criteria comprised the following:

(i) Studies unrelated to crop protection or pest management;
(ii) Articles focusing exclusively on synthetic pesticide development without a connection to agri-food-derived bioactive compounds;
(iii) Conference proceedings, editorials, patents, and non-peer-reviewed documents;
(iv) Publications lacking sufficient methodological description or reproducible experimental detail.

### 2.3. Study Selection Process

All records retrieved from the databases were exported and duplicates were removed prior to screening. Titles and abstracts were independently screened for relevance to the review scope. The full texts of articles meeting the preliminary criteria were subsequently assessed to confirm their eligibility.

Studies were selected based on their relevance to chemical diversity, mechanism of action, biological performance, and potential contribution to circular economy strategies. When necessary, the reference lists of eligible articles were manually screened to identify additional pertinent studies.

### 2.4. Data Extraction and Qualitative Assessment

Data were extracted focusing on: sources of agri-food residue, types of bioactive compound, extraction approach, reported biological activity, target organisms, and any discussion regarding environmental safety or regulatory considerations.

Although no quantitative meta-analysis was conducted due to heterogeneity among experimental designs, particular attention was given to methodological robustness, clarity in reporting biological assays, and the consistency of results. This qualitative appraisal aimed to ensure a critical and balanced interpretation of the available evidence.

## 3. Literature Search Outcomes and Study Selection

The structured database search yielded a total of 578 records. After the removal of 118 duplicate entries, 460 articles remained for title and abstract screening.

During the initial screening phase, 250 records were excluded for not meeting the predefined scope of the review, which was primarily due to the lack of direct relevance to agri-food waste valorization or absence of application in agricultural pest management.

A total of 210 reports were subsequently sought for full-text retrieval. All identified reports were successfully retrieved and assessed for eligibility. Following full-text evaluation, the 210 studies met the inclusion criteria and were incorporated into the qualitative synthesis.

**Figure 2:**
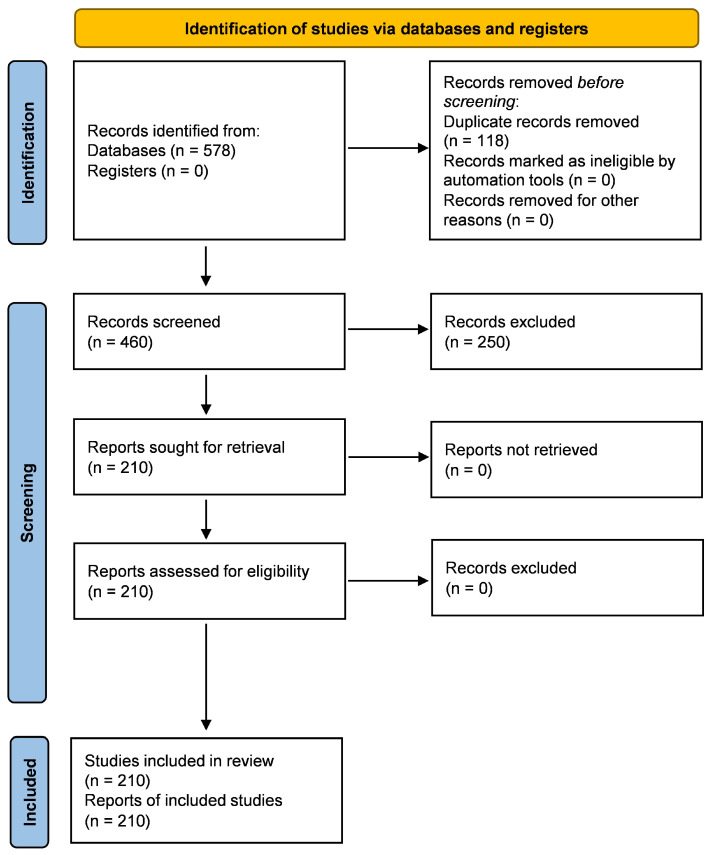
PRISMA flow diagram illustrating the literature search and study selection process. A total of 578 records were identified through database searching, of which 210 studies were included in the qualitative synthesis.

The PRISMA flow diagram (Figure 2) illustrates the stepwise selection process from the initial identification to the final inclusion.

## 4. Biopesticides: Definition, Classifications and Mechanisms of Action

The demand for safer, sustainable alternatives to synthetic pesticides has led to growing interest in phytopharmaceuticals of natural origin, particularly biopesticides. The global biopesticide market exceeded US$4 billion at the beginning of this decade, yet their registration in Europe remains low, around 68 products, which is mainly due to their strict regulatory structure, while the U.S. has approved over 400 products [16,20]. According to the US EPA, biopesticides are grouped into microbial pesticides, plant-incorporated protectants (PIPs), and biochemical pesticides, with the latter being the focus of this review [21]. Biochemical pesticides are derived from natural sources such as plant extracts, essential oils, and pheromones. They act via non-toxic mechanisms and can be sustainably obtained from agro-industrial by-products [21].

**Figure 3:**
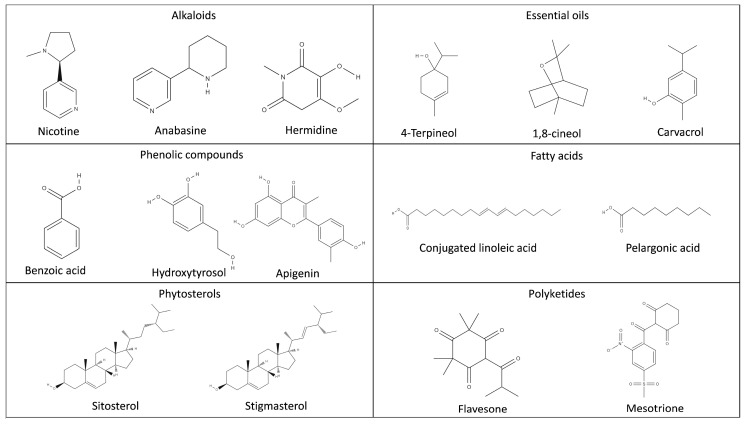
Chemical structure of bioactive compounds, mostly obtained from by-products and bio-wastes from agri-food industry [25,26,27,28,29,30].

These compounds originate from secondary plant metabolites, which are produced naturally by plants as defense agents against pests and pathogens. They can be classified according to both their functional groups and mechanisms of action, encompassing categories such as alkaloids, flavonoids, phenolic compounds, phytosterols, essential oils, fatty acids, polyketides, and resins [22,23,24]. The phytochemical diversity and biopesticidal potential of these molecules are illustrated in Figure 3, which highlights the chemical classes that are most frequently associated with pesticidal activity. They are particularly noted for their low environmental persistence and lack of bioaccumulation [16]. Within this context, various subgroups of biopesticides have been developed, each tailored to specific pest types.

Bioinsecticides, for instance, are designed to reduce the impact of insects responsible for 10–28% of global agricultural losses, according to the Food and Agriculture Organization (FAO) [31]. They operate through mechanisms such as feeding deterrence, inhibition of oviposition, disruption of growth and development, and neurotoxicity, often through interference with the octopamine receptors or GABA channels [32,33,34]. Compounds like azadirachtin and various essential oils exhibit significant repellent or antifeedant activities [32]. Nevertheless, the introduction of such products to the European market remains slow and is often difficult due to regulatory obstacles, as in the case of Bayer’s Lizetan AF [16]. Despite this, only about 1% of known secondary metabolites have been evaluated for insecticidal activity, suggesting a vast unexplored potential, as shown in Table 1. Bioherbicides focus on weeds, which are responsible for nearly 31.5% of global production losses: an estimated US$32 billion annually [35,36]. These products function through diverse mechanisms: inhibition of DNA synthesis, disruption of mitochondrial respiration, accumulation of reactive oxygen species (ROS), impairment of photosynthesis, and even microtubule destabilization [35,36,37]. Allelopathic plants offer selective or non-selective compounds with potent herbicidal properties, and several commercial formulations based on eugenol or pelargonic acid (e.g., Weed Slayer and Bio-Unkrautfrei AF) are already in use [37]. Biofungicides are vital in combatting fungal pathogens like *Fusarium*, *Aspergillus*, and *Penicillium* spp., which are responsible for ~30% of postharvest losses, existing in approximately 20,000 phytopathogenic fungi [37]. Their mechanisms include cell wall and membrane disruption, ROS induction, and reduction in aflatoxin production, among others [38,39,40,41,42]. Although fewer products have reached the market, ongoing research continues to reveal promising bioactive candidates. Biobactericides are designed to combat over 100 species of pathogenic bacteria affecting crops. These organisms, such as *Pseudomonas*, *Ralstonia*, and *Xanthomonas*, damage plants via toxin production, protein injection, or enzymatic degradation of tissues [43,44,45]. Natural compounds with biobactericidal activity damage the membrane integrity, respiration, and other vital bacterial processes [43,44,45,46]. Finally, bionematicides target nematodes, microscopic soil organisms that can cause crop losses, estimated at US$8 billion annually [43]. They interfere with root function, leading to stunted growth or death. Natural compounds targeting nematodes offer a promising alternative, as summarized in Table 1.

Together, these categories emphasize the wide-reaching potential of plant-derived biopesticides in supporting sustainable agriculture through eco-friendly and effective pest control solutions.

### Regulatory Challenges for Biopesticides: A Comparison Between the EU and the US

In the EU, biopesticides are evaluated and registered by following a process similar to that for synthetic ones, with legislation varying between countries. Consequently, the commercialization of new products is typically time-consuming and costly for the producer. However, certain requirements and criteria are irrelevant for this class, as they generally exhibit low toxicity to both the environment and humans.

In the United States, regulation of this product class is conducted by the US EPA, through the Biopesticides and Pollution Prevention Division (BPPD), based on a different set of requirements from those for synthetics [56,57]. The first step involves submitting a consultation request to the authorities, followed by a formal application to the BPPD, leading to an initial documentation review followed by a preliminary technical review [56,57]. If no issues are found at this stage, a scientific review concerning toxicological and ecotoxicological parameters and their physicochemical properties is conducted, with each category of biopesticides assessed accordingly [56,58]. If no problems are identified during the scientific review, the product obtains its biopesticide registration. The process typically takes around 12 to 18 months, with low financial fees involved [56].

It should be emphasized that, despite being derived from natural sources, biochemical biopesticides are not exempt from rigorous safety evaluation. Regulatory authorities require detailed toxicological, ecotoxicological, and environmental persistence and transformation data to assess the potential risks for non-target organisms, including pollinators, aquatic species, soil microbiota, and mammals [56,58]. Parameters such as acute and chronic toxicity, persistence, degradation pathways, and residue behavior must be carefully examined before approval. This regulatory investigation reflects the recognition that a natural origin does not inherently preclude adverse biological effects, and that risk assessment remains a central pillar in ensuring safe and sustainable implementation.

**Figure 4:**
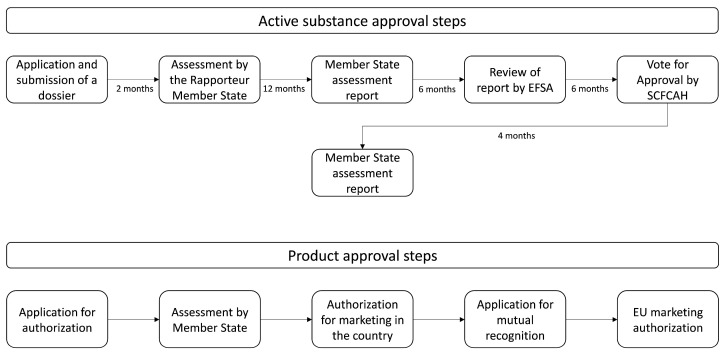
Stages of the approval process for an active substance and, later, for the marketing authorization of the product in the EU products [64].

In the EU, these product groups are covered by EU Plant Protection Regulation No. 1107/2009 together with Regulation No. 396/2005 and Directive 2009/128/EC [59,60,61]. The term “biopesticide” is not included in any European regulation, as Regulation No. 1432/2017 introduced the terms “basic substances” and “low-risk substances” [61,62]. Product release begins with the approval of its active substance, requiring the applicant to submit a dossier to the Rapporteur Member State containing information on the physicochemical, toxicological, and ecotoxicological properties, as observed in Figure 4 [59,60,61,62]. This dossier is assessed, and if there are no inconsistencies, the evaluation of the active substance begins. Upon completion, the Member State issues an assessment report of the substance to the European Commission and The European Food Safety Authority (EFSA) for their review [15,59,60,61,62]. If no inconsistencies are observed, the European Commission issues a review report to the Standing Committee on Plants, Animals, Food, and Feed (SCFCAH), conducting a vote for approval or rejection. This process can take between 30 and 42 months, depending on its complexity [15,59,60,61,62]. Due to this rigorous authorization process, there are currently about 18 basic substances and 10 low-risk substances released for use [63]. Low-risk active substances have an initial approval of 15 years with a review for an additional 15 years, while basic substances theoretically have approval for an unlimited period [15,59,60,61,62]. After approval, authorization for product marketing must be requested and conducted by the Member States, with the applicant specifying which Member State will carry out this evaluation, providing some product information, as shown in the flowchart in Figure 3 [15,59,60,61,62]. It is worth noting that for just one product, multiple dossiers may be required, as the product may contain more than one active substance.

## 5. Agri-Food By-Products as Rich Sources of Bioactive Compounds for Biopesticide Applications

The scarcity of natural resources coupled with environmental pollution has raised global awareness about the need to address, or at least mitigate, this issue due to its negative consequences. The agri-food industry is a major contributor, generating large amounts of organic waste and consuming scarce natural resources such as freshwater. It is crucial to develop new technologies for the reuse of these waste materials.

### 5.1. Olive-Oil By-Products

Olive oil is a widely consumed commodity globally, owing to its numerous health benefits and its critical role as an ingredient in other industries such as cosmetics. The olive tree belongs to the Oleaceae family, with *Olea europaea* L. being the only species capable of producing fruits that are suitable for olive oil production [65]. According to data released by the International Olive Council (IOC), the EU accounts for approximately 70% of the world’s olive oil production, with a market value of US$13.496 billion worldwide in 2024/25, with Spain being the largest producer [66]. It is noteworthy that this market has tripled in the last 60 years, reaching a production of 3.6 million tons in 2024/25 [67].

Olive oil production begins with the maintenance and care of olive trees, including pruning, weeding, pesticide application, and others [65,68]. Concurrently with harvesting, olives undergo a pre-cleaning process, generating branches (both thin and thick), leaves, and wood, which remain in the field. Subsequently, the olives are briefly stored and transported to mills. At the mills, olives are washed again to remove impurities and foreign materials, such as leaves, stones, damaged olives, and soil, among others [65]. Then, the olives undergo milling to break the stones, peel cells, and pulp (releasing vacuoles containing oil droplets), producing a homogeneous paste with stone fragments [65]. This paste is then transported to the malaxation phase to undergo physical and biochemical phenomena, correlated with the product’s quality and nutritional properties [65,69]. The paste proceeds to the separation phase, which can occur through three different processes: pressing and centrifugation in two or three phases [65,69]. Finally, various olive oil grades are obtained, ranging from extra virgin to lampante, generating olive mill wastewater, olive stones, and pomace.

The reviewed studies indicate that the olive oil extraction process generates large quantities of waste, with 80% of the olive weight turning into residues [70,71]. The major compounds in olive oil are fatty acids, followed by lignans and triterpenoids, whereas olive oil by-products contain high concentrations of phenolics, flavonoids, and secoiridoids, as shown in Table 2, along with low concentrations of tocopherols, fatty acids, and phytosterols, among others.

Regarding leaves and branches, it is estimated that pruning/harvesting annually generates a significant amount of olive by-products, about 25 kg per tree or between 1.5- and 3-tons ha^−1^, requiring further research to improve this value [69,72]. Most of this biomass is used for energy generation through incinerators and for animal feed [68]. Olive stones, constituting 10–15% of the weight of the olive, and still produced in the table olive industry, are commonly used in domestic boilers and power plants [88]. Another use is in the creation of a biodegradable packaging called Oliplast, as part of the Spanish project GO-OLIVA [89]. Olive mill wastewater is contingent on oil extraction processes, ranging from 85 to 110 kg for two-phase centrifugation to 1000–1200 kg for three-phase centrifugation [65]. The dispersion of this biowaste into the environment without prior treatment will cause significant issues due to high concentrations of toxic organic substances (chemical oxygen demand (COD) ranging from 50 to 200 g L^−1^ and biochemical oxygen demand (BOD) ranging from 40 to 170 g L^−1^), and a relatively acidic pH, altering the color and odor of water resources, and presenting toxicity to aquatic organisms [65,74,90]. Olive pomace production varies between 200 and 400 kg for pressing and 800–950 kg for two-phase centrifugation, featuring a pinkish paste-like appearance and slightly acidic pH (pH ≈ 5); it is composed of husk pieces, pulp, stone fragments and seeds, oil, and water [42,65,91]. This biowaste is the most abundant, generating approximately 14.4 million tons in the 2024/25 harvest season [66,92]. The pomace can be transported to other companies (pomace oil extractors), stored in large containers for spontaneous evaporation, and stored for extended periods until reused or treated [65,93]. The increase in olive oil production is correlated with increased pomace, posing challenges to the industry, and complicating the continuous production of high-quality olive oil [94].

Table 2 shows that the by-products generated in olive oil production contain a wide range of bioactive compounds, with potential for use as biopesticides, as presented in Table 3.

### 5.2. Potato Processing Waste

The potato (*Solanum tuberosum* L.) ranks among humanity’s most vital food crops, serving as a staple for approximately 1.3 billion individuals [99]. Its global production has soared, reaching a record 390 million metric tons in 2024, with Asian nations dominating, accounting for roughly 50%, led by China and India [100]. Financial transactions surrounding this commodity amount to approximately US$111 billion [100]. Boasting around 5000 varieties, potatoes exhibit unparalleled genetic diversity compared to other cultivated species [99].

The processing industry associated with this crop ranks among the largest in the global food sector. Approximately one-third of the total production is consumed fresh, while the remainder undergoes processing to meet the demands of convenience and fast-food consumption, including but not limited to: frozen fries, wedges, chips, starch, dehydrated potatoes, mashed potatoes, and frozen foods [99]. In the EU, as of 2023, processed potatoes hold a market value of around €9.7 billion, constituting roughly 2.1% of the European food industry’s value [101]. Among all the processed products, French fries are the most important, generating a market value of approximately €5.9 billion [102].

About 30% of this tuber is discarded during harvesting and storage, due to quality standards such as size, appearance, and pest damage. These discarded tubers, with low added value, are commonly repurposed as animal feed [99]. During processing, a significant portion of potatoes is peeled, yielding a substantial amount of nutrient-rich wet peel paste, ranging from 15 to 40% of the fresh weight, depending on the peeling technique [103]. Additionally, this industry generates outer layers of pulp, as well as pulp and wastewater from starch extraction [104]. Annually, this processing industry produces between 70 and 140 million tons of peels and 800–2800 million m^3^ of wastewater [105]. The disposal of untreated waste poses significant environmental concerns due to its potential for microbial degradation, constraining the storage options [102]. This substantial amount of waste yields ample opportunities for reuse, as potato peels are a source of bioactive compounds (starch, non-starch polysaccharides, proteins, antioxidants, or fibers, among others), as shown in Table 4. These compounds exhibit high recovery potential and find utility across various industries, fostering a more favorable economic balance between industrial processes and the commercialization of new products [99,106]. The concentration of these bioactive compounds in the waste depends on the potato variety as well as the agronomic conditions during cultivation and maturation [102,103].

Potato peels serve as a source of phenolic compounds, harboring approximately 50% of the compounds found in the potato. Investigations conducted by Brahmi et al., 2022 [113], reported the total phenolic content (TPC) in peels as ranging from 11 to 2840 gallic acid equivalent (GAE) mg 100 g^−1^, and total flavonoid content (TFC) varying from 780 to 2970 quercetin equivalent (QE) mg 100 g^−1^. In studies by Gomez-Urios et al. 2023 [114] utilizing UHPLC-MS/MS, 12 phenolic compounds were identified, wherein chlorogenic and trans-cinnamic acids were identified as being the most abundant compounds in free form in peels.

In addition to phenolic compounds, potato peels are also excellent sources of steroidal alkaloids, such as glycoalkaloids (α-solanine and α-chaconine) and aglycone alkaloids (solanidine and demissidine), as shown in Table 4, with α-solanine and α-chaconine glycosides and solanidine constituting about 95% of the total concentration [115].

Some of the applications of the phytochemical compounds obtained from potato waste are shown in Table 5.

### 5.3. Banana Waste

Banana stands out as one of the most popular and consumed fruits worldwide, due to its nutritional content and wide array of uses. It is the second most produced fruit worldwide [120,121] and belongs to the Musaceae family, which includes several hybrids of the genus Musa, with studies suggesting its origin in tropical regions of South Asia [122]. Global average production hovers around 120 million metric tons, whereas in 2023, the production reached 139 million metric tons, due to the significant delay in the reporting of the data, with India, China, and Indonesia being the top producers, accounting for approximately 40% of global output [100]. Reflecting its significance, the market value in 2023 approached US$140 billion [100].

Beyond fresh consumption, bananas find versatile applications, including dried fruits, snacks, smoothies, ice creams, breads, flours, wines, and ingredients for functional foods [121]. Notably, there has been a recent surge in utilizing this fruit as a functional food ingredient due to its low digestibility of carbohydrates (both starch and non-starch), rendering it a highly compelling dietary staple [123].

Banana plants yield a fruit bunch only once in their lifetime, resulting in considerable waste generation from harvesting to post-processing [121]. For every ton of harvested bananas, approximately 100 kg of fruit are discarded, generating around 4 tons of waste, comprising 160 kg of stems, 480 kg of leaves, and 3 tons of pseudostems [124]. Pseudostems resemble a trunk and are composed of overlapping leaf sheaths that provide support to the plant [125]. Most of this waste is typically returned to the field, while in the processing industries, banana peels represent the most significant residue, accounting for about 35–50% of the fruit’s weight. Annually, approximately 8 million tons of these residues are generated in this industry, highlighting their considerable utilization potential [100]. In some instances, banana peels can be repurposed as organic fertilizer and animal feed, due to their low tannin content and high fiber content [126].

Banana peels, like other residues, exhibit high potential for reuse due to their richness in organic compounds, including lipids, fibers, carbohydrates, and proteins, serving as an excellent source of bioactive compounds, as shown in Table 6 [121,123,124]. Several studies have identified over 40 compounds, categorized into four subgroups: phenolic acids, flavonols, flavan-3-ols (the largest subgroup), and catecholamines [123]. Moreover, banana peels demonstrate significant radical scavenging activity and reducing capacity compared to avocado, papaya, passion fruit, watermelon, and melon [127].

These bioactive compounds exhibit significant potential for various applications, including the agri-food, cellulose and paper, energy, fiber, and bioplastic industries [123,126,132]. Investigations by Ardila et al., 2024 [133], produced very high crystalline cellulose indices (up to 67.9%) from the extraction of pseudostem fibers through ultrasound-assisted extraction with NaOH. These produced fibers are comparable to, or even higher, than industrial fibers in terms of crystalline structure and cellulose content [133]. Particularly, these fibers, especially from the pseudostem, are being utilized as reinforcement in epoxy composites and raw materials in the textile industry [134]. Banana stems are used as flavorings [125].

The reviewed studies summarized in Table 7 present the latest applications and explore properties of biologically active compounds from banana waste in the agri-food industry, such as fertilizers or biopesticides.

### 5.4. Winery Waste

Grapes, which are commonly used, originate from approximately 60 different species within the Vitis genus, with *Vitis vinifera* L. being the most cultivated species, with production of approximately 77.7 million tons in 2024 [138]. Leading producers include China, Italy, and the United States, accounting for approximately 39% [139,140]. Given its significance, the market value of this product varies between US$550.5 billion [138]. Besides fresh consumption, a wide range of other forms exists, including wine, vinegar, juice, jam, jelly, dried, and seed oil [141,142].

The viticulture industry stands out as one of the most significant processing sectors, with more than 50% of all grape production allocated to winemaking [141,143]. According to data from the International Organisation of Vine and Wine (OIV), wine production reached approximately 225.6 million hectoliters in 2024, with major producers including European and American countries such as Italy, France, Spain, and USA [144]. The market value of wine from 2024 varies between US$500 and 520 billion [138,144]. This production spans across all countries worldwide, reflecting its profound cultural and socioeconomic significance [145]. The wine industry, while significant, poses considerable environmental concerns due to its high generation of waste and by-products. Negative impacts stem from improper management practices, low pH levels, the toxic effects of phytotoxic compounds, and the antimicrobial effects of residues [141,146]. Generated residues include vine shoots, grape pomace, wine lees, filtration cakes, vinasse, and winery wastewater, necessitating proper treatment for environmentally sound disposal [147]. Consequently, recent research investigates sustainable reuse options to maximize the utilization of these raw materials and enhance their value, given their rich content of bioactive compounds [148].

In wine production, approximately 20–30% of grape mass is transformed into pomace, consisting of 25% seeds, 25% stalks, and 50% other residues (broken pulp cells and grape skin), generated during crushing and pressing to obtain grape must [149,150]. This constitutes the primary solid residue of vinification, representing about 75% of all solid residues in the process [151,152]. It is estimated that producing approximately 6 L of wine generates about 1 kg of grape pomace, with annual production averaging 12 million tons of pomace [153]. Grape pomace comprises about 55–75% water, 30% polysaccharides, 6–15% proteins, lipids, sugars, and unsaturated fatty acids, with a high concentration of bioactive compounds, as shown in Table 8 [154]. Grape skin contains fibers, proteins, sugars, anthocyanins, flavonols, and tannins, with red grape skin potentially containing stilbenes (resveratrol), triterpenes, and derivatives of hydroxybenzoic acids [150,154]. The stalks consist of fibers such as cellulose, hemicellulose, lignin, and phenolic compounds like tannins [150,155]. Lastly, seeds, representing 2–5% of the weight, contain 40% fiber, 10% proteins, 10–20% lipids (mix of saturated and unsaturated fatty acids) and a wide range of phenolic compounds, sugars, and minerals [150,154,155].

Grape processing residues contain a high number of phytochemical compounds of significant interest to various sectors. Waste from wine production contains various phenolic compounds, with higher concentrations of anthocyanins, hydroxybenzoic and hydroxycinnamic acids, flavan-3-ols, flavonols, and stilbenes [169]. Montagner et al. 2022 [170] conducted hydroalcoholic extractions of crushed Merlot grape seeds, yielding high concentrations of bioactive compounds, with TPC ranging from 418.30 to 1473.86 μg GAE mL^−1^ of extract, flavonoids from 387.08 to 1000.63 μg catechin equivalent (CE) mL^−1^, and catechins from 0.14 to 0.59%. Ferreira and Santos, 2022 [152], investigated solid–liquid extractions with grape pomace and seeds, obtaining phenolic compound yields of 18.40% and 17.40%, respectively, and lipid yields of 13.30% and 14.50%, respectively. Phenolic extracts exhibited higher antioxidant capacity and DPPH^●^ scavenging assay than oils, with values of 90.80 and 87.50, respectively [152]. Regarding the antimicrobial capacity, phenolic extracts completely inhibited the growth of *S. aureus* and *S. epidermidis* [152].

Examples of potential applications in the agri-food industry, along with valuable phytochemical properties, are listed in Table 9.

### 5.5. Citrus Waste

Citrus fruits, belonging to the Rutaceae family, encompass a total of 27 different species, with the most important species being orange (*Citrus sinensis*), tangerine/mandarin (*Citrus reticulate*), lemon (*Citrus limon*) and lime (*Citrus aurantiifolia*) [176,177]. The cultivation origin of these fruits is uncertain, with one widely accepted theory suggesting that they have been cultivated for at least 4000 years in tropical and subtropical regions of Asia [178]. However, research by Rouseff et al. 2009 [179] has demonstrated that sweet orange originated in India, while trifoliate orange, along with tangerine, originated in China, and various other fruits have their origins in Malaysia.

Citrus fruits had a production of 170 million tons in the 2024 season, according to the World Citrus Organisation (WCO), with oranges (69 million tons), mandarins/tangerines (52 million tons), and lemons and limes (23 million tons) being the most prominent [180]. China leads citrus production, accounting for approximately 27.3%, followed by Brazil and India with 12.5% and 8.6%, respectively [180]. In the 2024 harvest, these fruits held significant commercial value, estimated at US$145–148 billion [180]. Besides fresh consumption, citrus fruits are found in juices, preserves, jams, marmalades, kitchen seasonings, and essential oils, among other products. Approximately 40% of the global production is utilized by the processing industry, primarily for juice production [181].

Overall, the reviewed studies suggest the processing industry of these foods generates a significant amount of solid waste, with approximately 50–65% of the fresh mass turning into residue obtained from fruit squeezing [176,182]. It is estimated that around 100–120 million tons of waste are produced annually, containing peel (60–65%), pulp (30–35%), and seeds (0–10%) [182,183]. These residues may also include portions of spoiled fruit [184]. Due to rapid decay and the appearance of flies, mold, and mycotoxins, these residues are perishable, posing serious disposal problems and requiring significant investments for citrus farming [181]. A major issue with this waste is that it cannot be disposed of in the environment without prior treatment due to its pollutant potential, necessitating landfill disposal, which is costly for industry. To mitigate these problems, processing industries often reuse these residues to produce cattle feed in pellet form, although such a product is not profitable [185]. These residues can still be repurposed in other sectors due to their high levels of soluble sugars, pectin, proteins, hemicelluloses, cellulose fibers, and bioactive compounds, especially flavonoids, and are also a valuable source of essential oil, as shown in Table 10 [186].

Due to its diverse chemical composition, this residue still holds potential for use in a range of biological activities, demonstrating that it is a valuable natural resource with added value [177]. One of its major uses is for the production of essential oils, with D-limonene representing about 94% of the composition, which is utilized in various industries, including agri-food, cosmetics, and pharmaceuticals, among others [177]. Furthermore, citrus residues, as shown in Table 10, still contain high amounts of organic and phenolic acids, also including some flavonoids, such as polymethoxylated flavones that are not found in any other fruit species [177,185]. The most studied polymethoxylated flavones are tangeretin and nobiletin, where studies by Lv et al. 2021 [194] found that nobiletin and its derivatives showed anticancer activity. However, such compounds still have a wide range of other biological activities, including anti-obesity, anti-atherosclerotic, antiviral, and antioxidant properties [195].

A summary of the main applications as a biopesticide, followed by its properties and results, is presented in Table 11.

## 6. Circular Economy and Industry 4.0: Integrating Biopesticide Production into Sustainable Agri-Food Systems

Previous studies on biopesticides derived from natural sources highlight the significant potential of agro-industrial residues and by-products for bioactive compound extraction and biopesticide production. Contrary to common practice, FAO distinguishes between “waste” (consumer-level) and “loss” (production and processing), aligning with circular economy principles, as well as the SDGs of the UN agenda 2030 [201]. The circular economy model fundamentally shifts from discarding materials and waste to reuse, repair, and recycling, creating a closed-loop production system, avoiding the disposal of consumed goods in landfills [202]. This model aims to minimize the use of new raw materials and the generation of waste and pollution. While some research focuses on using these materials for biofuel production, such as methane or ethanol, according to the “waste hierarchy”, utilizing these materials for energy production is less preferable compared to other strategies [202,203]. This is due to the high-value bioactive compounds that are still present in these residues and by-products. Another form of underutilization of these materials, although superior to their use for biofuel production, is in the production of feed and compost [203]. Countries such as Germany, France, and Italy have government initiatives for utilizing these residues, which are not suitable for human consumption, in the production of these products and have established regulations on this matter [203]. In terms of the SDGs, the use of these materials directly supports goals 9 and 12, promoting sustainable production and industrialization, and indirectly supports goals 14 and 15 by reducing the use of synthetic pesticides that are harmful to marine and terrestrial life [204]. Implementing these principles could reduce resource use by 17–24% by 2030, saving approximately €630 billion annually for European industries [64].

**Figure 5:**
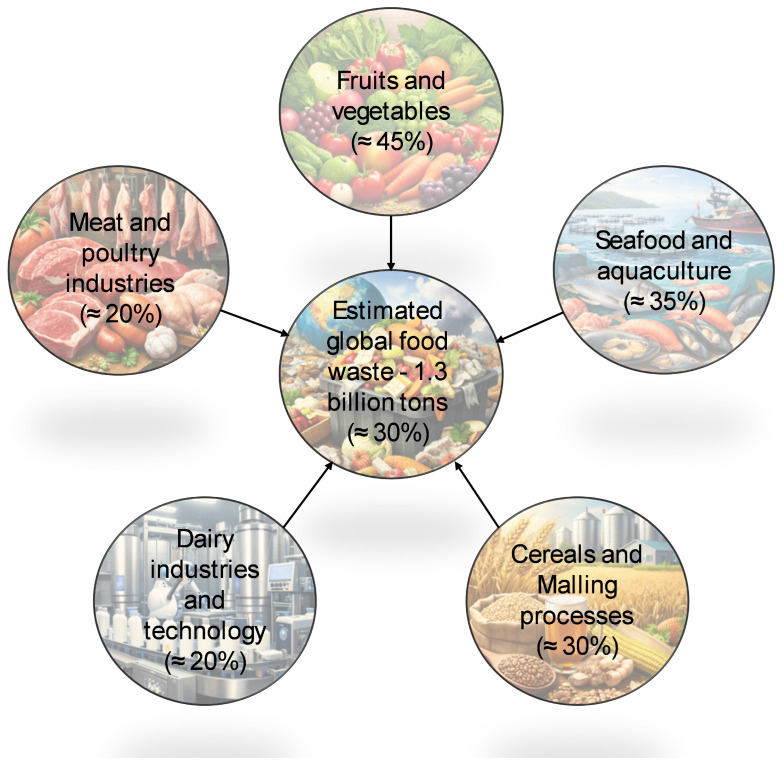
Pictorial representation of the total estimated annual food waste and loss across primary sectors.

Despite its importance, the transition to a circular economy, aligned with UN goals, encompasses not only the environmental and governmental aspects but also the social and economic factors at both industrial levels and in people’s daily lives. Socially, significant cultural changes in food consumption are needed, including reducing household waste and increasing the use of natural resources and reusable packaging [205]. Additionally, social inequality caused by poverty and hunger can be mitigated by producing high-quality, affordable food through the creation of businesses in collaboration with local industries, generating new jobs via circular technological systems [206]. These systems enable high-quality recycling and skilled jobs in transforming and remanufacturing agro-industrial losses, reducing raw material costs and boosting economic growth [205]. It is estimated that these practices could also increase employment by 4% and reduce greenhouse gas emissions by up to 70% [64]. Increased digitalization is radically transforming industrial infrastructures, making them connected, decentralized, and intelligent, in a development known as ‘Industry 4.0,’ driven by countries like Germany, the USA, and Japan [64]. Industry 4.0 is crucial for achieving greater efficiency, accuracy, and precision, utilizing technologies such as the Internet of Things (IoT), cloud manufacturing, and big data, among others. These advancements lead to stable production processes and the creation of new services and products. Industry 4.0 principles facilitate circular economy concepts by addressing economic uncertainties related to investment costs, financial returns, and implementation time, often due to a lack of information on product life cycles [207]. Additionally, few industrial plants currently focus on waste treatment and valorization [207]. The key elements of Industry 4.0 optimize circular economy business models by collecting and analyzing substantial data on material flows and energy consumption, supporting the generation of new value-added products [208]. In agri-food industries, Industry 4.0 implementation will reduce waste in various production stages, where 30–50% of food is lost, as shown in Figure 5, and enhance the valorization of residues and by-products through the development of new products.

Legal aspects related to food waste management must be considered according to each country’s laws. The EU aims to develop advanced recovery processes for incorporation into agri-food industries, surpassing traditional methods (animal feed, composting, anaerobic digestion), provided that the products are safe for human consumption and environmentally non-toxic [209]. The Treaty on the Functioning of the European Union includes regulations classifying waste based on recovery and reuse, with agro-industrial waste listed as non-infectious and suitable for incorporation into other processes [210]. However, as previously noted, certain regulations need to be adapted to facilitate quicker and simpler reuse of these materials, such as for biopesticide production.

## 7. Conclusions

Despite stricter controls on pesticide use and handling, the environment continues to receive a high load of these chemicals, leading to significant environmental and human impacts. As a result, there is a growing need to discover new products with low toxicity and minimal associated risks. Based on the evidence synthesized in this systematic review, one potential solution is the use of biopesticides, which generally only target specific species without accumulating in the environment or causing adverse effects, and without increasing species resistance due to their reduced persistence and residuality.

These products can be produced using bio-waste from the agri-food industry, which contains a wide range of valuable industrial phytochemicals, thus adding value to these residues instead of incurring treatment costs. The findings of this systematic review further indicate that European legislation for these products needs to be adapted to facilitate the market introduction of new products in a shorter timeframe and at lower costs, while greater international standardization of regulatory requirements is necessary to ease information exchange.

## Figures and Tables

**Table 1:** Potential sources of bioactive compounds and their diverse applications, including biopesticide action.

Compounds	Source	Organism	Application	Ref.
Alkaloids	*Ryania speciosa*	Insect	Alkaloids act by disrupting insect muscles by binding to calcium channels, causing ion influx and insect death, though their drawback is moderate mammalian toxicity.	[32]
Essential oil	*Foeniculum* *vulgare*	Insect	Ovicidal action against *Anopheles* spp. is linked to eugenol and cinnamaldehyde, while other phytochemicals inhibit cytochrome P450 or act as GABA-targeting neurotoxins.	[34]
Phenolic compounds	*Ipomoea cairica*	Insect	Larvicidal activity against *A. aegypti* is attributed to coumarins (7-hydroxychromen-2-one and 7-hydroxy-6-methoxychromen-2-one) and synergistic effects.	[47]
Essential oil	*Cinnamomum verum*	Weeds	Synergy between cinnamic aldehyde and eugenol manipulates metabolic pathways, showing bioherbicidal potential against *A. retroflexus*, *P. minor*, and *T. officinale*.	[48]
Monoterpenes	*Eucalyptus camaldulensis*	Weeds	1,8-cineole-rich extract disrupted membranes, exhibiting herbicidal activity against *P. sativum*, *A. repens*, and *P. oleracea*, enhanced by other terpenes.	[49]
Fatty acid	*Pelargonium graveolens*	Weeds	Pelargonic acid-rich extract showed bioherbicidal activity by inhibiting cellular and mitochondrial respiration in *A. fatua*, *Chenopodium* spp., and *P. oleracea*	[16]
Phenolic compounds	Mango residue	Fungi	Extract exceeds thiabendazole against *C. Brevisporum* micellar growth and spore germination, by disrupting membranes and inactivating enzymes.	[50]
Terpenes	*Thymus* *kotschyanus*	Fungi	Thymol, γ-terpinene, and carvacrol inhibited *B. cinerea* and partially-to-fully suppressed *A. niger* and *P. expansum* at 250–500 ppm via membrane disruption.	[51]
Essential oil	Orange peel	Fungi	D-limonene-rich EO showed superior biofungicidal activity, outperforming benomyl and quercetogetin in inhibiting mycelial growth.	[30]
Essential oil	*Satureja* *nabateorum*	Bacteria	EO showed stronger antibacterial activity than ampicillin (MIC = 0.14–2.25 vs. 1.00–3.12 µg mL^−1^) against *S. aureus*, *K. pneumoniae*, *E. faecium*, and *E. coli*, attributed to thymol-, γ-terpinene-, and p-cymene synergy.	[52]
Bioactive compounds	*Pleurotus* *ostreatus*	Bacteria	Mushroom extracts inhibited Gram-positive and Gram-negative bacteria via diverse secondary metabolites, due to phenolics, terpenes, phytosterols, etc.	[53]
Flavonoids	*Sophora exigua*	Bacteria	Extract inhibited MRSA via sophoraflavanone G and naringenin, reducing *E. coli*, *B. subtilis*, and *S. aureus* (200–400 µg mL^−1^) by decreasing membrane fluidity.	[44]
Flavonoids	*Rheum* *rhabarbarum*	Nematode	Laboratory and field studies reduced *M. javanica* infection in *T. aestivum*, *S. lycopersicum*, and *O. sativa*, attributed to catechin- and quercetin-rich extracts.	[54]
Phenolic compounds	*Momordica* *dioca*	Nematode	The extract showed strong bionematicidal activity against *H. indica*, causing 100% mortality with an LC_50_ of 17.80 mg mL^−1^, outperforming ivermectin (LC_50_ = 111.20 mg mL^−1^).	[55]

GABA: γ-aminobutyric acid, EO: essential oil, MIC: minimum inhibitory concentration, MRSA: methicillin-resistant *Staphylococcus aureus*, and LC_50_: lethal concentration 50%.

**Table 2:** Bioactive compounds are present in bio-waste from extraction process of olive oil.

Group	Bioactive Compounds	Concentration (mg kg^−1^ DW)	Ref.
Phenolic acids			
Hydroxycinnamic acids	Ferulic acid	0.01–12.60	[72,73,74]
	*p*-Coumaric acid	2.10–808.36	
	*o*-Coumaric acid	0.07–1.56	
	Caffeic acid	2.89–1830.00	
Hydroxybenzoic acids	Syringic acid	0.29–0.73	[75,76,77]
	Vanillic acid	26.50–170.23	
	Gallic acid	ND–61.00	
	Protocatechuic acid	1.30–136.70	
	4-Hydroxybenzoic acid	1.75–13.80	
Flavonoids			
Flavonols	Rutin	0.14–48.52	[74,78,79]
	Quercetin	ND–0.76	
Flavones	Luteolin	0.01–510.00	[74,80,81,82]
	Luteolin-7-glucoside	0.09–597.90	
	Luteolin-4′-*O*-glucoside	0.01–0.48	
	Luteolin-hexoside	0.01–24.20	
	Apigenin	0.33–9.55	
	Apigenin-7-*O*-glucoside	0.01–343.70	
	Apigenin-7-*O*-rutinoside	0.70–0.90	
Secoiridoids and derivatives			
–	Oleuropein	2.82–230.70	[74,83,84,85,86,87]
	Hydroxytyrosol	0.35–23,842.00	
	Tyrosol	0.42–21,190.00	
	Comselogoside	6.00–11,242.70	
	Verbascoside	0.57–1588.90	
	Oleacein	14.56–7698.91	
	Oleocanthal	66.39–3596.98	
	Pinoresinol	1.40–630.00	

DW: dry weight and ND: non-detected.

**Table 3:** Potential applications for biopesticides of phytochemical compounds obtained from olive oil by-products.

Residue	Extraction Method	Bioactive Compounds	Application	Ref.
Olive leaves	Extracted with ethanol/water (50:50) by 6 h at SLR 1:20 at RT with stirring method.	40 compounds identified, with ↑ concentration for secologanoside and oleuropein derivates.	Extract exhibited ↑ antimicrobial activity with MIC = 50 mg mL^−1^ for *E. coli*, *S. enterica* and *S. aureus* and inhibition of *P. aeruginosa* (70%) and *B. cereus* (67%).	[95]
Olive leaves	Methanol by 5 h at SLR 1:5 at RT (3 times), followed by an extraction with ethyl acetate.	Oleuropein: 215.26 and 958.22 mg g^−1^.	MIC range: 50–0.781 mg mL^−1^, with *S. aureus* being the most sensitive and *E. coli* the least.	[96]
OMWW	Liquid–liquid extraction with pure ethyl acetate, ethanol or methanol.	TPC = 2.16 g GAE L^−1^ (ethyl acetate), 2.97 g GAE L^−1^ (ethanol), 4.03 g GAE L^−1^ (methanol).	Methanol extract exhibited the ↑ antimicrobial activities on all 10 bacterial strains, with *S. aureus* being the most sensitive and *C. albicans* the least.	[97]
OMWW	Crude residue was concentrated by microfiltration, reverse osmosis, and membrane distillation.	Antioxidant activity (DPPH^•^-SA) = 10–80% and TPC = 1.5–15 g GAE L^−1^.	All samples analyzed showed antibacterial activity against Gram-positive and Gram-negative pathogens, affecting the growth of *P. syringae* pv. *tomato*.	[98]
Olive pomace	Heat-assisted extraction: water/ethanol (24:76), for 120 min and at 85 °C.	Yield = 13.70% and TPC = 148.88 mg g^−1^, with ↑ tyrosol and HYT derivates.	Isolated compounds and extracts served as food preservatives, offering alternatives to synthetic additives and potential health benefits.	[85]
Olive pomace	Pressing force patent process (PCT/IB2018/060111) with 4 varieties of olives.	TPC = 3.05–3.83 g GAE 100 g^−1^, TFC = 1.96–3.17 g CE 100 g^−1^, hydroxytyrosol: 63.33–220 mg 100 g^−1^.	Extract exhibited ↑ antimicrobial activity for *E. coli* (MIC = 62.5 mg mL^−1^) and *S. aureus* (MIC = 31.25 mg mL^−1^), but not for *C. albicans*.	[42]

OMWW: olive mill wastewater, RT: room temperature, SLR: solid–liquid ratio, MIC: minimum inhibitory concentration, TPC: total phenolic content, GAE: gallic acid equivalent, DPPH^•^-SA: 2,2-diphenyl-1-picrylhydrazyl radical scavenging, TFC: total flavonoids content, CE: catechin equivalent, and HYT: hydroxytyrosol.

**Table 4:** Bioactive compounds present in residues from potato processing industry.

Group	Bioactive Compounds	Concentration	Ref.
Phenolic acids			
Hydroxycinnamic acids ^(a)^	Chlorogenic acid	1.17–7.91	[105,107]
	Caffeic acid	0.25–5.21	
	Ferulic acid	0.04–0.86	
	*p*-Coumaric acid	0.01–0.12	
Alkaloids			
Glycoalkaloids ^(b)^	α-Chaconine	873–4014	[107,108,109]
	α-Solanine	597–3229	
	Solanidine	374	
	Demissidine	75	
	Total glycoalkaloids	6.71–3580	
Flavonoids			
Flavonols ^(a)^	Rutin	0.05–5.00	[107,110]
	Quercetin	2.18–11.22	
	Catechin	5.0–12.0	
Anthocyanins ^(c)^	Delphinidin	0.49–2.48	[111,112]
	Cyanidin	0.25–7.17	
	Petunidin	0.40–203.22	
	Pelargonidin	0.99–143.05	
	Peonidin	0.56–55.97	
	Malvidin	0.08–28.71	

^(a)^ Expressed in mg 100 g^−1^ dry weight, ^(b)^ expressed in mg kg^−1^ dry weight, and ^(c)^ expressed in mg 100 g^−1^ fresh weight.

**Table 5:** Potential applications for biopesticides of phytochemical compounds obtained from potato residues.

Residues	Extraction Method	Bioactive Compounds	Application	Ref.
Potato leaves	Extract: dichloromethane, ethyl acetate, and ethanol, overnight at RT and constant agitation.	TLC analysis suggested the identification of terpenoid and aromatic compound.	Extract inhibited the mycelial growth of *B. cinerea* with an ED_50_ of 4.3 mg L^−1^.	[116]
Potato peels	Extraction with ethanol overnight in a shaker at RT, and the residue was re-extracted 3 times.	TPC: 22–49 mg GAE g^−1^ and DPPH^•^-SA: 51–65% inhibition.	Biofilms showed ↑ inhibition of *E. coli*, *S. enterica*, and *S. aureus*; however, a negative response for *K. pneumoniae* and *L. monocytogenes*.	[117]
Potato peels	Peel powder was mixed with water, filtered, treated with NaOH to separate starch, and dried at 40 °C for 24 h.	Starch was enzymatically converted into isomaltose-rich IMOs via sequential α-amylase and α-glucosidase treatments.	Purified isomaltose, from the peels, showed ↑ antifungal activity against *E. cichoracearum* (concentration = 1.0 mg mL^−1^) and *F. oxysporum* (2.5 mg mL^−1^).	[118]
Potato peels	Soxhlet extraction: 70% ethanol, at boiling point of ethanol for 48 h, followed by a concentration.	16 bioactive compounds were identified, including l-verbenone and pyrogallol.	Extract completely inhibited fungal growth of *A. niger* and *A. flavus* at 250 mg mL^−1^ at mango roots.	[119]

RT: room temperature, TLC: thin-layer chromatographic, ED_50_: median effective dose, TPC: total phenolic content, GAE: gallic acid equivalent, DPPH^•^-SA: 2,2-diphenyl-1-picrylhydrazyl radical scavenging, and IMOs: isomalto-oligosaccharides.

**Table 6:** Bioactive compounds present in residues from banana waste.

Group	Bioactive Compounds	Concentration (mg kg^−1^ DW)	Ref.
Phenolic acids			
Hydroxycinnamic acids	Ferulic acid	6.00–212.48	[128,129]
	Sinapic acid	1.02–3.07	
	*p*-Coumaric acid	1.93–11.20	
Flavonoids			
Flavonols	Rutin	242.2–618.7	[123,128,130]
	Kaempferol	9.30–173.90	
	Myricetin	22.50–115.20	
	Quercetin	6.14–72.50	
Flavan-3-ols	(+)-Catechin ^(a)^	1.34	[123,131]
	Epicatechin ^(a)^	2.55–5.97	
	Gallocatechin	42.00–158.00	
	Procyanidin B1 ^(a)^	1.27	
	Procyanidin B2 ^(a)^	81.95	
	Procyanidin B4 ^(a)^	7.90	
Catecholamines			
–	Dopamine ^(b)^	86.56–205.56	[120,123]
	*L*-Dopa ^(c)^	0.31–0.56	

^(a)^ Expressed in molar percentage, ^(b)^ expressed in ppm dry weight, and ^(c)^ expressed in mg g^−1^ dry weight.

**Table 7:** Potential applications for biopesticides and fertilizers of phytochemical compounds obtained from banana waste.

Residue	Extraction Method	Bioactive Compounds	Application	Ref.
Banana peels	Methanol for 3 days, filtered and evaporated in a rotary evaporator at 60 °C.	7 phenolics and 3 flavonoids were identified, ↑ rutin (973.08 mg 100 g^−1^ DW), ellagic acid, etc.	↑ antibacterial (*A. tumefaciens*, 90 mm IZ) and antifungal activity (*F. culmorum*, 68.88%; *R. solani*, 94.07%).	[128]
Banana peels	Ethanol, acetone, or methanol (SLR 1:10–20) by maceration (40 °C, 20 h) or sonication (35–55 °C, 1 h).	50% ethanol yielded 13.48% extract, with TPC = 31.46 mg GAE g^−1^ DW and TFC = 22.11 mg QE g^−1^ DW.	50% ethanol sonication extracts (600 ppm) inhibited *S. aureus*, *P. aeruginosa*, *E. coli*, and *S. cerevisiae* (11.31–15.43 mm IZ).	[135]
Banana peels	Peels were mixed with sugar and fresh curd in water and fermented for 15 days in airtight conditions.	EDX analysis of the biofertilizer revealed high levels of oxygen, silicon, and iron.	Biofertilizer ↑ black grass germination in a concentration-dependent manner, reaching 100% at 20 mL L^−1^ within 7 days (vs. 12% control).	[136]
Banana peels	Peels were KOH-treated for 30 min, filtered, pH-adjusted to 5, and dried at 105 °C.	Biofertilizer particles (19–55 nm) contained chelated K and Fe, tryptophan, urea, amino acids, proteins, and citric acid.	Germination increased dose-dependently, reaching 97% in tomato and 93.14% in fenugreek after 7 days (vs. 14% and 25% controls).	[137]

SLR: solid–liquid ratio, TPC: total phenolic content, TFC: total flavonoids content, GAE: gallic acid equivalent, QE: quercetin equivalent, DW: dry weight, and IZ: inhibition zone, EDX: energy dispersive X-ray.

**Table 8:** Bioactive compounds present in residues from winery waste.

Group	Bioactive Compounds	Concentration (mg kg^−1^ DW)	Ref.
Phenolic acids			
Hydroxybenzoic acids	Gallic acid	25.2–360.4	[156,157,158]
	Ellagic acid	2.8–36.3	
	Protocatechuic acid	9–63	
	Vanillic acid	10–230	
	Syringic acid	469–1731	
Hydroxycinnamic acids	Caffeic acid	0.41–82.8	[156,157,159,160]
	Caftaric acid	~200	
	*cis*-Coutaric acid	5.30–40.00	
	*trans*-Coutaric acid	5.50–40.00	
	*p*-Coumaric acid	2.85–77.4	
Flavonoids			
Flavonols	Quercetin	3–200	[157,161,162,163,164,165]
	Quercetin-3-*O*-glucoside	67.60–3274.30	
	Rutin	0.11–8.19 ^(a)^	
	Myricetin	0.21–2.31 ^(a)^	
	Kaempferol	2.45–53.10 ^(a)^	
	Quercitrin	0.21–3.99 ^(a)^	
Flavanols	Catechin	43.1–3711.00	[160]
	Epicatechin	12.3–189.00	
Flavones	Apigenin	0.02–12.7 ^(a)^	[157]
	Luteolin	0.23–1.07 ^(a)^	
Anthocyanins	Delphinidin 3-*O*-glucoside	0.15–1.86 ^(c)^	[166,167]
	Cyanidin 3-*O*-glucoside	0.13–0.79 ^(c)^	
	Petunidin-3-*O*-glucoside	0.21–2.96 ^(c)^	
	Malvidin-3-glucoside	0.99–8.94 ^(c)^	
	Peonidin-3-*O*-glucoside	0.45–3.58 ^(c)^	
Fatty acids			
–	Palmitic acid	5.5–9.2 ^(b)^	[168]
	Oleic acid	10.8–24.9 ^(b)^	
	Palmitoleic acid	ND–0.6 ^(b)^	
	Linoleic acid	60.9–78.2 ^(b)^	
	Linolenic acid	0.2–0.6 ^(b)^	

^(a)^ Expressed in mg kg^−1^ FW, ^(b)^ expressed in % of fatty acids extracted from grape pomace, ND: non detected, and ^(c)^ expressed in µg mL^−1^ FW.

**Table 9:** Potential applications for biopesticides of phytochemical compounds obtained from winery residues.

Residues	Extraction Method	Bioactive Compounds	Application	Ref.
Grape pomace	Ground material was pyrolyzed at 350 or 700 °C for 2 h under N_2_ (10 °C min^−1^), yielding BC350/700 and washed forms.	Pyrolysis ↑ C and nutrients, reduced carboxyl groups, alkalinized biochars, and produced porous, mineral-rich structures favorable for microorganisms.	Washed biochar (0.75%, BC350W) significantly reduced *M. javanica* infection and reproduction in tomato under controlled conditions.	[171]
Grape pomace	Extracted 4 times with acidified methanol (0.1% HCl) at RT for 4 h, 12 h, 4 h, and 12 h intervals.	Extract retained high phenolics (5–35 mg GAE/g DW), especially fermented Syrah and Alicante seeds, rich in flavan-3-ols and procyanidins.	Natural antioxidants for use in functional foods or to improve product stability and shelf life.	[172]
Grape pomace	Overnight at ethanol (96%) with SLR 1:10 in stirring method.	No identification and quantification of the compounds.	Extract showed ↑, variety-dependent antimicrobial activity, especially against *B. subtilis*, with synergistic effects alongside antibiotics.	[173]
Skins and seeds	70% acetone/water overnight, filtered, and dried in a forced-air oven at 60 °C for 96 h.	No identification and quantification of the compounds.	Extract showed antimicrobial activity against Gram-negative (*E. coli*) and Gram-positive (*B. subtilis*) bacteria.	[174]
Grape pomace	Soxhlet extraction with methanol for 8 h.	GC-MS and LC-MS identified major fatty acid esters and phenolics in grape pomace, including ethyl linoleate and trans-stilbene	Nanoencapsulated extract showed antimicrobial-related protection, providing sustained pest control efficacy and enhanced stability in a single application	[175]

RT: room temperature, GAE: gallic acid equivalent, DW: dry weight, and SLR: solid–liquid ratio.

**Table 10:** Bioactive compounds present in residues from citrus waste.

Group	Bioactive Compounds	Concentration (mg kg^−1^ DW)	Ref.
Organic acids			
–	Citric acid	19,487–27,810	[187]
	Lactic acid	5563–9961	
	L-malic acid	3046–5164	
Phenolic acids			
Hydroxybenzoic acids	Gallic acid	8.74–856.70	[187,188]
	Protocatechuic acid	24.45–65.92	
	4-Hydroxybenzoic acid	25.27–41.50	
Hydroxycinnamic acids	Ferulic acid	19.50–139.60	[187,189,190,191]
	*p*-Coumaric acid	18.20–243.40	
	Chlorogenic acid	0.08–68.78	
	Caffeic acid	4.10–1325.10	
Flavonoids			
Flavones	Apigenin	58.91–158.67	[187,191]
	Vitexin	32.73–119.27	
	Luteolin	92.47–276.14	
Flavanones	Hesperidin	2316.50–21,486.00	[188,190]
	Naringin	9.20–19,550.00	
	Narirutin	63–10,442	
Volatile compounds	*β*-Linalool	379.50–14,610	[188,190]
	*β*-Myrcene	819–3216	
	*β*-Ocimene	360–2860	
Carotenoids			
–	Lutein	0.76–28.89	[192,193]
	*β*-Carotene	1.10–36.62	

**Table 11:** Potential applications for biopesticides of phytochemical compounds obtained from citrus waste.

Residue	Extraction Method	Bioactive Compounds	Application	Ref.
Citrus by-products	Maceration 3 times with ethanol (20%) for 72 h each, with filtration and solvent removal after each cycle.	14–16 phenolic compounds identified in all residues, ↑ concentrations of rutin, myricetin, sinapic and ferulic acid.	↑ antibacterial activity against Gram-positive bacteria and for Gram-negative. ↑ antifungal activity against *C. albicans*, *A. flavus*, *A. niger* and *F. oxysporum*.	[196]
*Citrus aurantifolia*	Extraction with hot water for 15 min at SLR 1:15, 2 times.	Active fractions yielded known coumarins, limonoids, flavonoid glycosides, and aurantifolin.	Chloroform fraction showed the highest toxicity against *B. tabaci* (LC_50_ = 37.1) nymphs, outperforming butanol fraction and azadirachtin after 24–72 h.	[197]
*Citrus reticulata*	Pilot unit for discontinuous extraction with ethanol (96%) at SLR 1:2 for 15 h.	Extract had diverse secondary metabolites, dominated by limonene (~70%), with flavonoids, tannins, and steroids also present.	2.5% formulation controlled pests (*F. occidentalis* and *Aphididae*) comparably to chemicals, spared beneficial insects, and achieved 73% of conventional crop yields.	[198]
*C. sinensis*, *C. aurantium* and *C. reticulata* peels	Peels were removed, dried for 15 days, and subjected to 4 h hydrodistillation.	13–16 different compounds were identified, ↑ concentrations for D-limonene, terpinene and myrcene.	Extracts inhibited seed germination and seedling growth, with complete suppression of *H. annuus* at all tested concentrations	[199]
Lemon, orange, and grapefruit peel	Extraction by piercing peel in water with collection via water spray, or hydrodistillation for 3 h, then drying.	Compounds were quantified, dominated by limonene, β-myrcene, and α/β-pinene in lemon and grapefruit essential oils.	Orange extract was most effective against *R. dominica*, *Oryzaephilus* sp. and *S. granarius*. Antifungal activity: *R. solanii* was more susceptible than *S. rolfsii*, with lemon and orange being the most toxic.	[200]

SLR: solid–liquid ratio and LC_50_: lethal concentration 50.

## Data Availability

No new data were created or analyzed in this study.

## Abbreviations

The following abbreviations are used in this manuscript:

BPPD	Biopesticides and Pollution Prevention Division
CE	Catechin equivalent
COD	Chemical oxygen demand
BOD	Biochemical oxygen demand
DDT	Dichlorodiphenyltrichloroethane
DPPH^●^	2,2-diphenyl-1-picrylhydrazyl radical scavenging
DW	Dry weight
EDX	Energy dispersive X-ray
EFSA	European Food Safety Authority
EO	Essential oil
EU	European Union
FAO	Food and Agriculture Organization
GABA	γ-aminobutyric acid
GAE	Gallic acid equivalent
IOC	International Olive Council
IoT	Internet of Things
LC_50_	Lethal concentration 50
MIC	Minimum inhibitory concentration
MRSA	Methicillin-resistant *Staphylococcus aureus*
OIV	International Organisation of Vine and Wine
PIPs	Plant-incorporated protectants
PRISMA	Preferred Reporting Items for Systematic Reviews and Meta-Analyses
QE	Quercetin equivalent
ROS	Reactive oxygen species
SCFCAH	Standing Committee on Plants, Animals, Food, and Feed
SDHI	Succinate dehydrogenase inhibitor
SDGs	Sustainable Development Goals
SLR	Solid–liquid ratio
TPC	Total phenolic content
UN	United Nations
US EPA	United States Environmental Protection Agency
WCO	World Citrus Organization
